# Annexin A1 downregulates *in vitro* IL-1*β* production in *L. braziliensis*-infected cells

**DOI:** 10.3389/fimmu.2025.1685264

**Published:** 2025-10-22

**Authors:** Camila P. Santos, Mauricio T. Nascimento, Marina B.R. Santana, Edgar M. Carvalho, Lucas P. Carvalho

**Affiliations:** ^1^ Laboratório de Pesquisas Clínicas, Instituto Gonçalo Moniz, FIOCRUZ, Salvador, Brazil; ^2^ Programa de Pós-Graduação em Ciências da Saúde, Universidade Federal da Bahia, Salvador, Brazil; ^3^ Serviço de Imunologia, Complexo Hospitalar Prof. Edgard Santos, Universidade Federal da Bahia, Salvador, Brazil; ^4^ Instituto Nacional de Ciências e Tecnologia-Doenças Tropiais, Salvador, Brazil

**Keywords:** Annexin A1, cutaneous leishmaniasis, *L. braziliensis*, immune response, inflammation

## Abstract

Cutaneous leishmaniasis (CL) is an infectious disease characterized by severe local inflammatory response, predominantly mediated by cytokines such as IL-1*β* and TNF, and cytotoxicity, contributing to tissue damage and lesion development. Given the high rate of therapeutic failure in *Leishmania braziliensis* transmission areas, the investigation of molecules that regulate inflammatory response has become a promising adjuvant therapeutical strategy. In this study we investigated the effects of Annexin A1 (ANXA1) on the inflammatory response of CL patients. We initially performed *in silico* analyses from our previous transcriptome databases and found increased expression of *ANXA1, IL1B*, and *IL10* genes in skin biopsies from CL patients when compared to healthy skin from healthy subjects (HS). Also, increased levels of ANXA1, IL1*β*, and IL10 proteins were observed in serum levels and cultures of skin biopsies in CL patients when compared to HS. Treatment of lesion biopsies with recombinant ANXA1 reduced IL-1*β* levels without affecting IL-10 secretion, indicating a selective anti-inflammatory effect. Additionally, monocyte-derived macrophages from HS increased ANXA1, IL-1*β*, and IL-10 production upon *Leishmania* infection. Blockade of FPR2 receptor increased ANXA1 levels. Finally, addition of recombinant ANXA1 to macrophages did not affect the ability of these cells to kill *Leishmania*. Our findings demonstrate that ANXA1 negatively regulates IL-1*β* in CL, without impairing anti-inflammatory mechanisms or macrophage microbicidal activity, highlighting its potential use as an adjuvant therapy for controlling inflammation and disease progression.

## 1 Introduction

Cutaneous leishmaniasis (CL) is the most common clinical form of American tegumentary leishmaniasis (ATL) and *Leishmania braziliensis* is the main etiological agent causing ATL in Brazil (1). CL is characterized by skin lesions with elevated borders, a granulomatous base, and an intense inflammatory infiltrate, predominantly composed of lymphocytes and mononuclear phagocytes, along with a reduced number of parasites (2, 3). Although the inflammatory response is associated with control of parasite replication, its exacerbation leads to the development of cutaneous ulcers. CL patients have production of Interleukin 1 beta (IL-1*β)* and decreased expression of the interleukin 10 (IL-10) receptor in lesions, which is associated with the inability to control pro-inflammatory effects, ultimately leading to tissue damage (4, 5).

In regions endemic for *L. braziliensis*, pentavalent antimonial (SbV) remains the first-line therapy recommended by the Brazilian public health system. However, high therapeutic failure rates, reaching up to 70% depending on the clinical form is documented. Additionally, patients in the pre-ulcerative stage (early cutaneous leishmaniasis, ECL) are more likely to experience treatment failure with SbV (6, 7). Thus, there is an urgent need for new adjuvant therapies capable of reducing inflammation and parasite replication, thereby minimizing treatment failure in CL.

Annexin A1 (ANXA1) is a 37 kDa protein belonging to the annexin superfamily. It is induced by glucocorticoids and expressed in the cytosol of myeloid, epithelial, and endothelial cells (8). After cell activation, ANXA1 is externalized via vesicles from the plasma membrane. When in the extracellular medium, it acquires a conformation (through its N-terminal region) to bind to the G protein-coupled receptor FPR2/ALX (9, 10). Annexin A1, already well-described in the literature as an inflammation regulator, when induced by glucocorticoids, it has been shown to be an inhibitor of phospholipase A2 (11). Its primary receptor, FPR2, acts as a crucial mediator of its anti-inflammatory properties and regulates leukocyte trafficking during the inflammatory response (12). Murine knockout models for ANXA1 exhibited exacerbated inflammatory responses and resistance to steroid anti-inflammatories (13). In another animal model, ANXA1 protected the myocardium against ischemia-reperfusion injury (14). Treatment with the ANXA1-derived mimetic peptide, Ac2-26, in models of endotoxin-induced uveitis, reduces leukocyte infiltration and the release of inflammatory mediators, including IL-1*β* (15). Also, in mice subjected to intestinal ischemia-reperfusion and treated with Ac2-26, increased levels of IL-10 and a reduction in plasma TNF were observed, suggesting a role for ANXA1 in promoting IL-10-mediated anti-inflammatory responses in experimental models (16).

Considering the central role of inflammation in the pathogenesis of CL and its association with tissue damage and therapeutic failure, it is essential to identify new strategies or adjuvant therapeutic targets to modulate the inflammatory response. In the present work we found that ANXA1 decreases IL-1*β* production, independent of IL-10, without affecting the ability of macrophages to kill *Leishmania* parasites.

## 2 Methods

### 2.1 *In silico* analysis

Transcriptomic data from skin and peripheral blood were obtained from GSE127831 (17) and GSE162760 (18), respectively, and labeled according to the provided metadata. The dataset included 21 skin samples from *L. braziliensis*-infected patients before treatment, 7 uninfected endemic controls, 50 peripheral blood samples from *L. braziliensis*-infected patients before treatment, and 14 uninfected endemic controls. Additionally, *L. braziliensis* sequenced by RNA-seq was analyzed. Gene expression levels of PYD and CARD domain containing *(PYCARD)*, caspase-1 *(CASP1)*, NLR family pyrin domain containing 3 *(NLRP3)*, *IL1B, IL10, FPR2*, and *ANXA1* were evaluated. Heatmaps and correlation matrices were generated using RStudio Cloud.

### 2.2 Subjects

This study was approved by the Research Ethics Committee of the Faculty of Medicine of Bahia (protocol number: 2471185) and the Brazilian National Research Ethics Commission (protocol number: 2512.434). All participants provided written informed consent. The study was conducted in accordance with the Declaration of Helsinki and its subsequent revisions. Fifty-eight CL patients were recruited from the endemic area of leishmaniasis, Corte de Pedra, Bahia, Brazil. The diagnostic criteria included the presence of an ulcerated skin lesion, without evidence of mucosal involvement, and the detection of *L. braziliensis* DNA by PCR (19, 20). The control group consisted of twenty-eight HS living in a non-endemic area of the same state, with no reported exposure to *Leishmania*. All CL patients underwent clinical evaluations before starting treatment.

### 2.3 Monocyte-derived human macrophage cultures and *L. braziliensis* infection

Monocyte-derived macrophages from HS were prepared from peripheral blood mononuclear cells (PBMCs) were isolated from heparinized venous blood by Ficoll-Paque (GE Healthcare, Chicago, IL, USA) gradient centrifugation. After being washed in saline, the cells’ concentration was adjusted to 2,5 x10^6^ cells in 0,5 ml of RPMI-1640 (low glucose) (Thermo Fisher Scientific, NY, USA) supplemented with 10% FBS, penicillin (100 U/ml), and streptomycin (100 *µ*g/ml) (Thermo Fisher Scientific, NY, USA). Monocytes were separated by adherence in labtek plates. After 6 days of culture, the adherent cells displayed characteristics of monocytes-derived macrophages. After differentiation of monocytes to macrophages, the cells were infected with stationary-phase *L. braziliensis* promastigotes at a 5:1 ratio for 4 hours, while uninfected macrophages served as controls. After incubation, the infected cells were cultured in the presence or absence of recombinant Annexin A1 (rAnnexin A1) (R& D Systems, Minneapolis, MN, USA) at concentrations of 1 *µ*g/ml, as well as WRW4 (Tocris Bioscience, Bristol, UK) (23 *µ*M), an FPR2 inhibitor, for 4 and 48 hours. At each time point, culture supernatants were collected and the levels of IL-1*β*, IL-10, and Annexin A1 were quantified by enzyme- linked immunosorbent assay (ELISA). Additionally, the infection rate and parasite load were assessed by three independent observers, through microscopy.

### 2.4 Biopsy culture

Biopsies of the active CL lesion and healthy skin from HS from lower limbs were performed using a 4 mm punch. Whole fragments were weighed and placed in tubes containing 1 mL of RPMI-1640(Gibco, Waltham, MA, USA) supplemented with 10% FBS (Gibco, Waltham, MA, USA), 100 U/ml penicillin, and 100 *µ*g/ml streptomycin, and incubated at 37 *°*C, 5% CO_2_ for 48 hours in the presence or absence of 1 µg of rAnnexin A1 (R&D Systems, Minneapolis, MN, USA).

### 2.5 Parasite culture

An isolate of *Leishmania braziliensis* (MHOM/BR/LTCP30833) was obtained from a skin lesion of a patient with CL and identified as *L. braziliensis* through multilocus enzyme electrophoresis (21, 22). After isolation, the parasites were cryopreserved in liquid nitrogen until use. For this study, they were thawed and expanded in culture only once in Schneider’s medium (Sigma-Aldrich, St. Louis, MO, USA) supplemented with 20% heat-inactivated fetal bovine serum (FBS), 1% L-glutamine, 100 U/ml penicillin, and 100 *µ*g/ml streptomycin (Thermo Fisher Scientific, New York, NY, USA). Parasites in the stationary phase were used for the experiments.

### 2.6 Cytokines quantification

The levels of IL-1*β*, IL-10 (BD Biosciences, San Diego, CA, USA) and Annexin A1 (RayBiotech, Inc., Norcross, CA, USA) were determined by ELISA in serum samples from CL patients and HS, as well as in supernatants from *L. braziliensis*-infected macrophage cultures. All assays were performed according to the manufacturers’ instructions.

### 2.7 Statistical analysis

Statistical analyses were performed using GraphPad Prism 9.0 and RStudio. Data were expressed as mean and standard deviation. Differences between groups were assessed using the Mann-Whitney or Wilcoxon test, as appropriate. Correlations were analyzed using Pearson’s test. Differences with p *≤* 0.05 were considered statistically significant.

## 3 Results

### 3.1 ANXA1 and inflammatory genes expression are increased in clinical samples from CL patients

**Figure 1:**
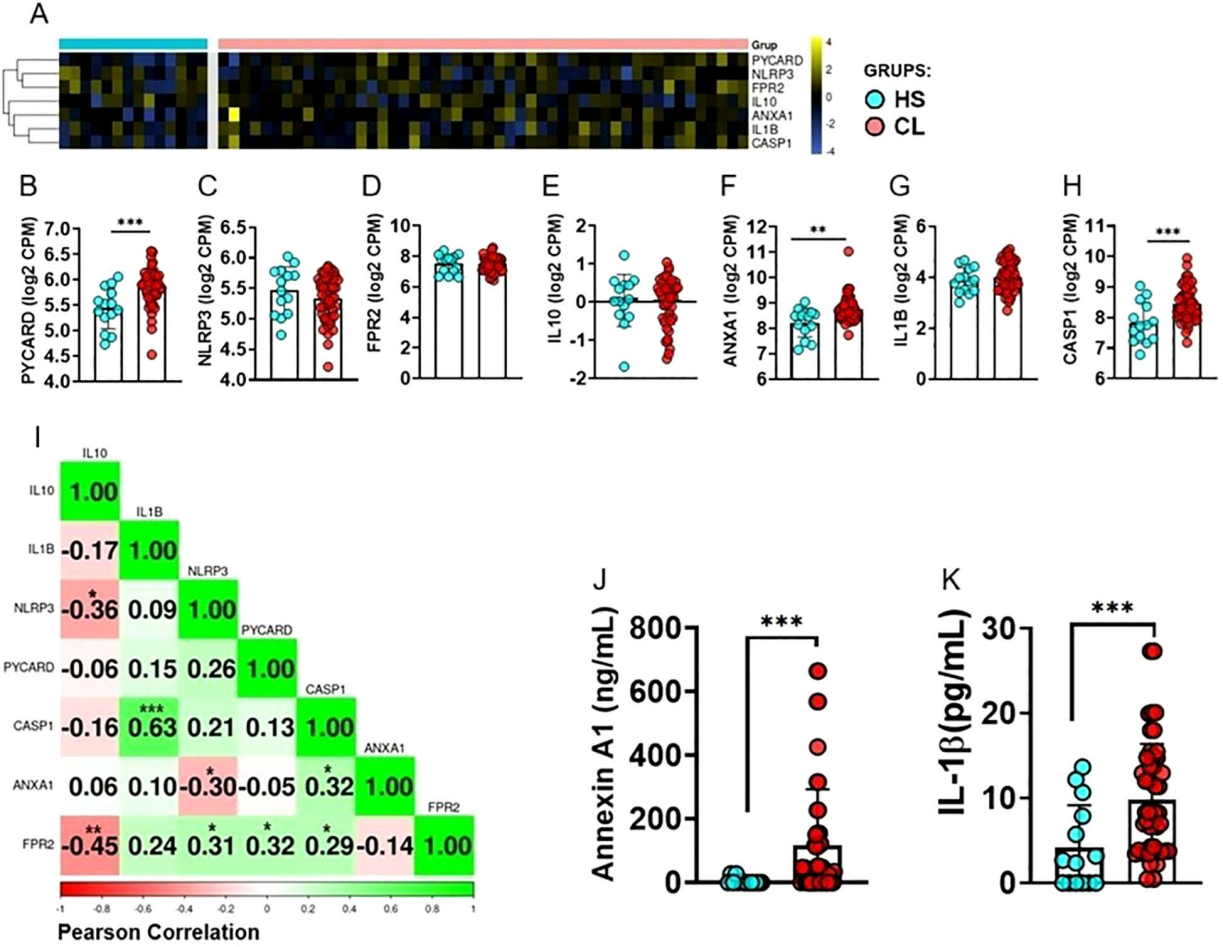
Differentially Expressed Genes in Clinical Samples from Cutaneous Leishmaniasis Patients. **(A)** Heatmap showing clustering of gene expression for *PYCARD, CASP1, NLRP3, IL-10, IL1B, ANXA1, and FPR2* in peripheral blood from HS (n = 14) and CL patients (n = 50). Rows represent genes and columns represent individuals. The color scale indicates relative expression levels, ranging from blue (low expression) to yellow (high expression). **(B-H)** Differences in gene expression between CL patients and HS for **(B)** *PYCARD*, **(C)** *NLRP3*, **(D)** *FPR2*, **(E)** *IL10*, **(F)** ANXA1, **(G)** *IL1B*, and **(H)** *CASP1. PYCARD, CASP1*, and *ANXA1* showed significantly higher expression in CL patients compared to HS. **(I)** Pearson correlation matrix of *PYCARD, NLRP3, CASP1, IL1B, FPR2*, and *ANXA1* expression in peripheral blood from CL patients (n = 50). Positive correlations are shown in green and negative correlations in red. **(J)** Serum levels of ANXA1 and **(K)** IL-1*β* in CL patients (n = 50) and HS (n = 14), determined by ELISA. Results are expressed as mean ± standard deviation. Statistical analysis was performed using the Mann–Whitney test. *P *<* 0.05, **P *<* 0.01, ***P *<* 0.001.

To investigate association between *ANXA1* and inflammasome pathways, we assessed gene expression in the peripheral blood from HS and CL patients. Our analysis revealed increased expression of *ANXA1*, *CASP1*, and *PYCARD* in the peripheral blood of CL patients, while there was no difference in the expression levels of *FPR2*, *NLRP3*, *IL1B*, and *IL10* (**Figures 1A-H**). Subsequently, we constructed a correlation matrix using the same genes but exclusively from CL patient samples. This analysis revealed a positive correlation among most genes, particularly between *FPR2* and *NLRP3*, *CASP1*, and *PYCARD*. In contrast, *ANXA1* negatively correlated with *NLRP3* (**Figure 1I**). We also investigated ANXA1 and IL-1*β* protein in blood. Despite negative correlation between *ANXA1* and *NLRP3* gene expression, we found increased serum levels of ANXA1 and IL-1*β* protein in CL patients when compared to HS (**Figures 1J, K**).

**Figure 2:**
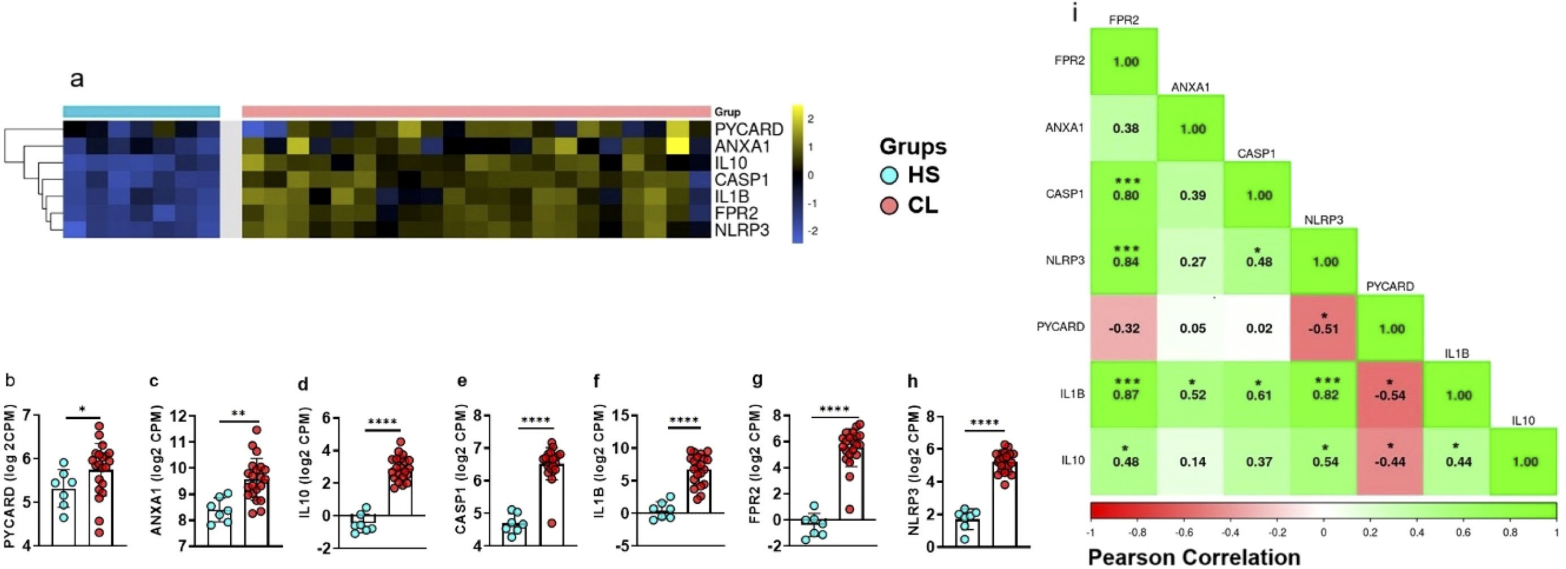
CL patients show higher expression of inflammasome-related genes in active lesions. **(A)** Heat map showing expression of *PYCARD*, *ANXA1*, *CASP1*, *IL1B*, *FPR2*, and *NLRP3* in lesion biopsies from CL patients (n = 21) and healthy skin samples (n = 7), clustered by similarity. Blue indicates low expression, yellow indicates high expression. **(B-H)** Gene expression differences between CL lesions and healthy skin for **(B)** *PYCARD*, **(C)** *NLRP3*, **(D)** *FPR2*, **(E)** *IL10*, **(F)** *ANXA1*, **(G)** *IL1B*, and **(H)** *CASP1*. **(I)** Correlation matrix for gene expression in CL lesions (n = 21) showing positive correlations (green) and negative correlations (red). Results are expressed as mean *±* standard deviation. Statistical analysis was performed using the Mann–Whitney U test for **(B-H)** and Pearson correlation for **(I)**. *P < 0.05, **P < 0.01, ***P < 0.001.

Systemic immunity may not reflect the immune response that happens at lesion site. Thus, we then assessed the expression of the genes studied above in HS skin and compared with those from CL lesion. Our data show significantly higher expressions of *PYCARD*, *ANXA1*, *CASP1*, *IL1B*, *FPR2*, *IL10*, and *NLRP3* in CL lesions when compared to HS (**Figures 2A-H**). We then generated a correlation matrix with the expression of the genes *PYCARD*, *CASP1*, *NLRP3*, *IL1B*, *ANXA1*, and *FPR2* from CL lesions. We identified positive correlations between inflammasome pathway genes (*NLRP3*, *CASP1*, and *IL1B*) and *FPR2*, as well as between *ANXA1* and *IL1B* (**Figure 2I**). Collectively, these results strengthen the idea that inflammasome activation is a major feature of CL pathogenesis, both at the systemic level and at the lesion site.

### 3.2 ANXA1 downregulates IL-1*β* production in cells from CL lesion

**Figure 3:**
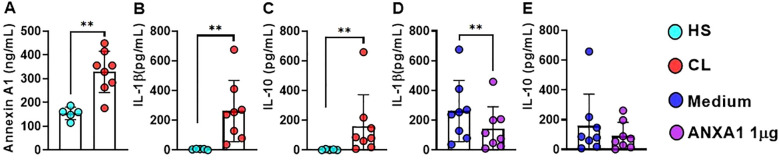
ANXA1 down-regulates IL-1*β* Production in CL. Levels of **(A)** ANXA1, **(B)** IL-1*β*, and **(C)** IL-10 determined by ELISA in supernatants from CL lesion biopsy cultures (n = 8) and healthy skin from HS (n = 5). **(D)** IL-1*β* and **(E)** IL-10 in lesion biopsy cultures (48 hours) from CL patients treated or not with rANXA1 (1 *µ*g). Results are expressed as mean *±* standard deviation. Statistical analysis was performed using the Wilcoxon test. **P < 0.01.

Since we found *ANXA1* gene expression in blood and lesion from CL patients, we then investigated whether ANXA1 protein would be present in CL lesions. Thus, we determined the concentration of ANXA1, IL-1*β* and IL-10 protein in the supernatant of HS skin and lesion border biopsies from CL patients. We found increased levels of ANXA1, IL-1*β* and IL-10 in cells from CL lesion cultures when compared to HS skin (**Figures 3A–C**). ANXA1 has been associated with both inflammation and immunoregulatory responses. To understand the effects of ANXA1 in CL, we treated CL biopsies cultures with recombinant ANXA1 (rANXA1) and determined IL-1*β* and IL-10 concentrations after 48 hours. We observed that the biopsies treated with rANXA1 exhibited significantly lower levels of IL-1*β* when compared to the untreated biopsies cultures, whereas no change was observed in IL-10 production (**Figures 3D, E**). This finding suggests that treatment with rANXA1 may exert a regulatory effect on IL-1*β* production in CL patients, independent of IL-10, supporting the idea that ANXA1 plays a role in modulating inflammation.

### 3.3 ANXA1 is produced by *L. braziliensis*-infected macrophages

**Figure 4:**
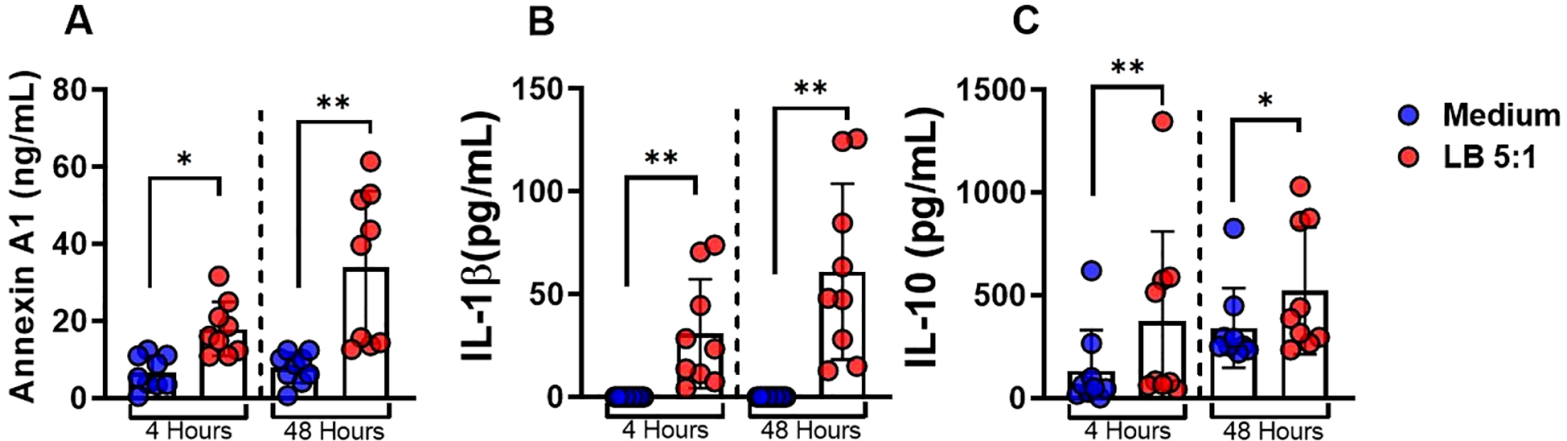
ANXA1 is produced by *L. braziliensis*-infected macrophages. Monocytes-derived macrophages were infected with *L. braziliensis* (5:1) for 4 and 48 hours. **(A)**ANXA1, **(B)** IL-1*β* and **(C)** IL-10 levels determined by ELISA. Results are expressed as mean *±* standard deviation. Statistical analysis was performed using the Wilcoxon test. *P < 0.05, **P < 0.01.

We found increased levels of ANXA1 in CL lesion. Since macrophages are the main cells parasitized by *Leishmania*, we asked whether *L. braziliensis*-infected macrophages produced ANXA1. For that we conducted assays using monocyte-derived macrophages (MDMs) from HS infected with *L. braziliensis* at a ratio of 5:1 (parasites:cell). Increase in the levels of ANXA1, IL-1*β*, and IL-10 were observed in the supernatant of *L. braziliensis*-infected cultures after 4 and 48 hours (**Figures 4A–C**).

### 3.4 ANXA1 downregulates IL-1*β* through the receptor FPR2 in *L. braziliensis*-infected macrophages

**Figure 5:**
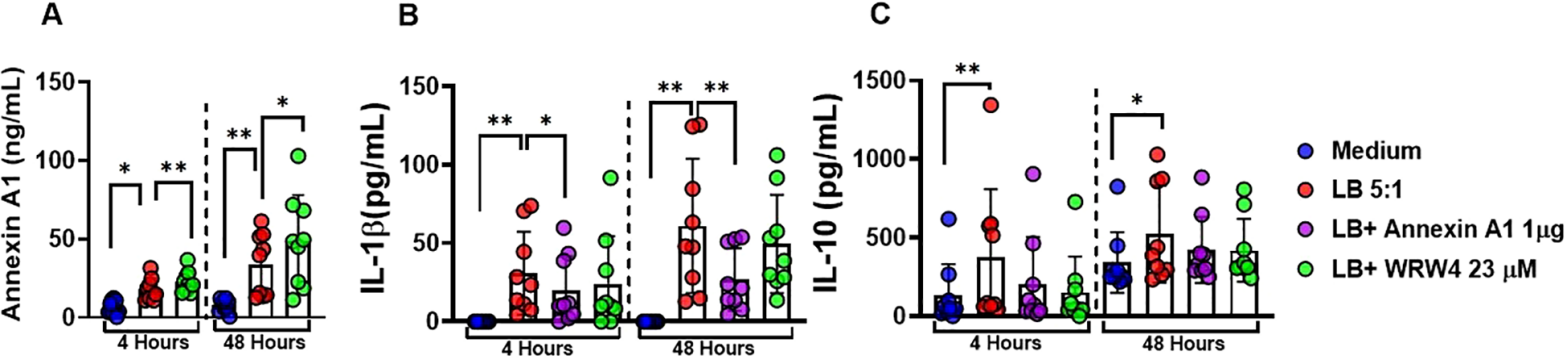
ANXA1 downregulates IL-1*β* through the receptor FPR2 in *L. braziliensis*-infected macrophages. Levels of ANXA1, IL-1*β*, and IL-10 in culture supernatants of monocyte-derived macrophages from healthy subjects (n = 9) infected with *Leishmania braziliensis* (5:1) in presence and absence of recombinant Annexin A1(1 *µ*g/mL) and WRW4(23 *µ*M/mL) at 4 and 48 hours. **(A)** ANXA1 levels, **(B)** IL-1*β* levels, and **(C)** IL-10 in culture supernatants were determined by ELISA. Results are expressed as mean *±* standard deviation. Statistical analysis was performed using the Wilcoxon test. *P < 0.05, **P < 0.01.

To investigate the role of ANXA1 in *Leishmania*-infected macrophages we cultured (MDMs) in the presence or absence of rANXA1 and WRW4, a selective inhibitor of the *FPR2* receptor, one of the main receptors for ANXA1. Addition of rANXA1 decreased IL-1*β* levels and blockade of FPR2 receptor abrogated its effect (**Figures 5A–C**). These findings show that annexin modulates IL-1*β* production through FPR2 receptor signaling in *L. braziliensis* infection in human macrophages. As expected, the addition of ANXA1 did not affect IL-10 production. These data suggest that ANXA1 regulates IL-1*β* production independent of IL-10.

### 3.5 ANXA1 does not affect phagocytic ability and parasite killing by macrophages infected with *L. braziliensis*

**Figure 6:**
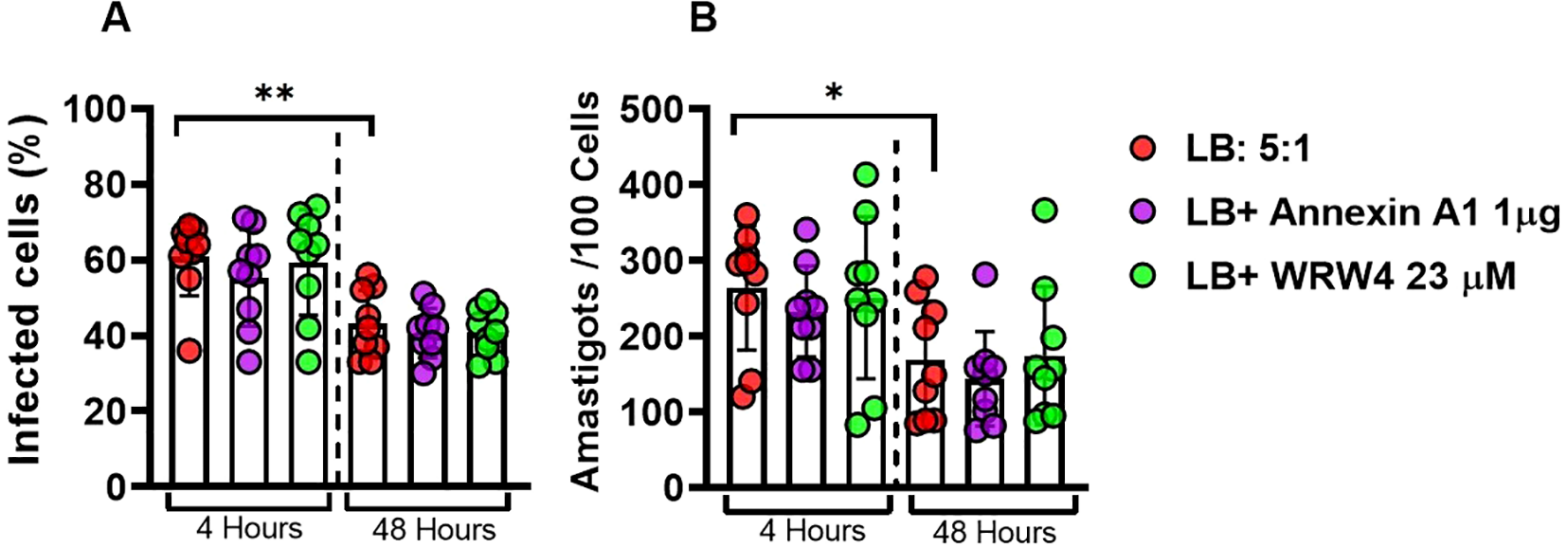
ANXA1 does not affect phagocytic ability and parasite killing by macrophages infected with *L. braziliensis*. Effect of WRW4 and rANXA1 treatment on monocyte-derived macrophages from healthy subjects (n = 9) infected with *Leishmania braziliensis* (5:1) at 4 and 48 hours. rate infection **(A)** and the number of amastigotes per 100 cells **(B)**. Results are expressed as mean *±* standard deviation. Statistical analysis was performed using the unpaired t-test. *P < 0.05, **P < 0.01.

Although IL-1*β* has not been associated with *L. braziliensis* killing in human macrophages, data on literature has documented a role for IL-1*β* in *Leishmania* parasites killing in animal models (23). Thus, we assessed whether the negative regulation of IL-1*β* promoted by ANXA1 would impair the phagocytic and killing ability of macrophages. As documented before, we observed a reduction in both the infection rate and number of parasites within macrophages over time (24). Moreover, the presence of ANXA1 or its receptor (*FPR2*) inhibitor showed no influence in the ability of macrophage to kill *Leishmania* parasites (**Figures 6A, B**).

## 4 Discussion

IL-1*β* is an inflammatory cytokine produced by macrophages and neutrophils that participate in the pathogenesis of various diseases, such as autoimmune, autoinflammatory, metabolic, and neurodegenerative diseases, contributing to the severity of the pathological process (25, 26). In parasitic diseases such as leishmaniasis, there is a complex interaction between different Leishmania species and the host immune system, mediated by cytokines such as IL-1*β* and IL-10, which determines the clinical outcomes of the disease. Each species manifests a distinct cytokine profile: while certain strains of *L. major* (such as LmSd) trigger a robust IL-1*β* response that contributes to lesion pathology, *L. amazonensis* suppresses the initial production of inflammatory cytokines, facilitating the establishment of the infection (27). *L. infantum*, in turn, induces both pro-inflammatory IL-1*β* and regulatory IL-10, reflecting the complexity of visceral leishmaniasis. In parallel, IL-10 is consistently associated with parasite persistence and immune dysregulation in infections with *L. amazonensis* and *L. major* (28, 29), while in *L. braziliensis* and *L. tropica* IL-1*β* is the main cytokine associated with tissue damage and its release depends on the activation of the NLRP3 inflammasome (23, 24, 30, 31). IL-1*β* not only has a significant expression but has also been associated with lesion size (32) and areas of lesion necrosis (24). IL-10 is seen as a cytokine with anti-inflammatory functions that regulates the immune response Oliveira et al. (33); Santos et al. (24); Lima-Junior et al. (23).

ANXA1 is a protein with anti-inflammatory properties and has been associated with downregulation of IL-1*β* production. Here we investigated the role of ANXA1 in IL-1*β* production in CL patients. Our results show that ANXA1 is produced by *L. braziliensis*-infected macrophages and in CL lesions, and by signaling through FPR2, ANXA1 decreases IL-1*β* production.

To investigate the role of ANXA1 in CL, we first analyzed gene expression in peripheral blood data from CL patients and identified a significant increase in the expression of *PYCARD*, *CASP1*, and *ANXA1* when compared to HS. These findings indicated that, in addition to the local inflammatory response, there is evidence of systemic inflammasome activation in CL patients. *PYCARD* and *CASP1* are core components of the inflammasome, essential for the activation of caspase-1 and the subsequent processing and release of IL-1*β* (34, 35). Thus, their systemic upregulation suggests the possibility of inflammasome formation and activation in the circulation, which may contribute to elevated systemic IL-1*β* levels and the exacerbation of inflammation in different compartments (23). Interestingly, the correlation matrix analysis of gene expression in the blood of CL patients revealed positive correlations between *FPR2* and *NLRP3*, *CASP1*, and *PYCARD*, suggesting that the FPR2-mediated pathway may play a role in modulating systemic inflammasome activation. On the other hand, we observed that *ANXA1* negatively correlated with *NLRP3*, which may indicate a potential regulatory role of ANXA1 in restraining NLRP3 inflammasome activation in peripheral blood, possibly attempting to control exacerbated inflammation.

We also analyzed gene expression at lesion site. The expression of *PYCARD*, *CASP1*, *NLRP3*, *IL1B*, *ANXA1*, and *FPR2* genes were higher in CL lesion samples when compared to those from skin of HS. This pattern sustains the idea of intense inflammasome activation within the lesion microenvironment, contributing to local IL-1*β* production (35). The correlation matrix generated from these data indicated that *FPR2* was positively correlated with *CASP1*, *NLRP3*, *IL1B*, and *IL10*, suggesting that, besides potentially favoring inflammasome activation, FPR2 may also be associated with a regulatory feedback mechanism, possibly mediated by IL-10. Our findings are consistent with studies reporting that FPR2 activation is associated with the modulation of inflammatory response, demonstrating that increased release of anti-inflammatory cytokines such as IL-10, particularly during the resolution phase, occurs when FPR2 agonists promote the suppression of pro-inflammatory mediators, while simultaneously enhancing the release of anti-inflammatory factors, including IL-10, decreasing inflammatory response and tissue repair (36). Notably, in lesion samples *ANXA1* positively correlated with *IL1B*, suggesting a possible dual role for this molecule, in both controlling and perpetuating the inflammatory response, depending on the tissue compartment. These findings raise several important questions, especially given the discrepancies among different authors’ reports. It is widely accepted that a relationship exists between ANXA1, IL-1*β*, and the NLRP3 inflammasome, and that ANXA1 and NLRP3 co-localize in the cytosol, indicating potential functional interaction (37–40).

Therefore, controversial studies have been published regarding the effects of ANXA1 on IL-1*β* production. For instance, it was reported that ANXA1 is required for IL-1*β* release in response to NLRP3 inflammasome activation. Using rodent macrophages, it was demonstrated that ANXA1 regulates the transcription and expression of *NLRP3*, *pro-IL-1β*, and *pro-caspase-1*, and directly interacts with NLRP3 in a FPR2- independent manner, suggesting its involvement in inflammasome assembly and activation (37). In contrast, it was shown that peritoneal macrophages from ANXA1-deficient rodents stimulated with nigericin or ATP exhibited an exacerbated release of IL-1*β* following NLRP3 inflammasome activation, suggesting that ANXA1 plays an anti-inflammatory modulatory role by limiting inflammasome activation (38). Supporting the last findings, in human keratinocytes stimulated under atopic dermatitis conditions, levels of both NLRP3 and ANXA1 were elevated, along with the release of IL-1*β*, and treatment with an ANXA1 mimetic peptide modulated IL-1*β* production and NLRP3 activation, highlighting the relevance of this interaction in cutaneous inflammation (40).

These divergences underscore the importance of considering cell type and inflammatory stimulus in each study, particularly in the context of CL. The results obtained in our study suggest a strong association between serum levels of ANXA1 and IL-1*β* and the inflammatory profile observed in CL. We observed that CL patients exhibit significantly higher levels of both ANXA1 and IL-1*β* in serum when compared to HS, reinforcing the hypothesis that ANXA1 may play an important role in modulating the systemic inflammatory response associated with infection. Similar mechanisms have been described in other chronic inflammatory diseases. In a murine model of gout, for instance, ANXA1 was shown to promote neutrophil apoptosis and acute inflammation resolution, being essential to control chronic inflammation associated with gout (41). Additionally, an *in vitro* and *in vivo* mouse transplant model investigating the role of ANXA1 in modulating monocyte migration, suggested that ANXA1 negatively regulates cell migration and chronic inflammation (42).

To better understand the local anti-inflammatory effects mediated by ANXA1, we performed 48-hour *ex vivo* cultures from lesion edge biopsies of CL patients and healthy skin from HS. The results showed that the levels of ANXA1, IL-1*β*, and IL-10 in culture supernatants from lesion edge biopsies were significantly higher when compared to those from healthy skin biopsies. Similar findings have been reported in another study demonstrated that the absence of ANXA1 results in greater susceptibility to infection, with exacerbated inflammatory responses, impaired control of parasitism, and reduced IL-10 levels Ricotta et al. (43) The increased levels of ANXA1 at CL lesion site suggests that this protein may be involved in modulating the inflammatory response, possibly acting as a regulatory mechanism to restrain exacerbated inflammation. Similar findings have been reported in other chronic inflammatory models, where ANXA1-deficient animals exhibited exacerbated inflammatory responses in carrageenan-induced edema and zymosan-induced peritonitis models, with increased leukocyte migration and IL-1*β* production (13, 44).

We also evaluated the effects of exogenous ANXA1 on cells from CL lesions. We observed that culture supernatants from treated biopsies showed significantly reduced IL-1*β* levels when compared to untreated cultures. Our results are consistent with previous work in an endotoxin-induced uveitis model in rodents and *in vitro* studies with human cells, where systemic and topical administration of ANXA1 resulted in decreased leukocyte infiltration in ocular tissues and reduced expression of inflammatory mediators (15).Similar findings have been reported in another study using a model of *L. amazonensis* infection demonstrating that the absence of ANXA1 results in greater susceptibility to infection, with exacerbated inflammatory responses, impaired control of parasitism, and reduced IL-10 levels (43). Also, in mice model of *L. braziliensis*, it was documented that ANXA1 downregulates inflammatory response and improves lesion resolution (45). The reduction of IL-1*β* observed in our study strengthened the potential immunoregulatory role of ANXA1 in attenuating the exacerbated inflammation, a characteristic of CL lesions.

In contrast, no significant differences were observed in IL-10 levels between treated and untreated cultures. Data available in literature indicate that the anti-inflammatory effects of ANXA1 may be related to IL-10. In animal models, ANXA1 administration promoted IL-10 production, while in IL-10-deficient animals, the anti-inflammatory effects of ANXA1 were abolished, suggesting an interdependence between these molecules (44). Our studies indicate that ANXA1 may selectively modulate pro-inflammatory mediators such as IL-1*β* without affecting the release of the anti-inflammatory cytokine IL-10, representing a strategic mechanism by which the host restrains exacerbated inflammation without compromising parasite control and resolution mechanisms.

Experiments with HS monocytes-derived macrophages infected with *L. braziliensis* revealed a multifaceted inflammatory regulation profile mediated by ANXA1, IL-1*β*, and IL-10, highlighting an interesting feedback mechanism involving the FPR2 receptor. When rANXA1 was added exogenously, a significant reduction in IL-1*β* levels was observed at both 4 and 48 hours, clearly demonstrating a regulatory effect on this pro-inflammatory cytokine. The fact that IL-10 levels remained unchanged under these conditions further supports the selectivity role of ANXA1. Intervention with WRW4, a selective inhibitor of the FPR2 receptor - one of the main receptors for ANXA1 - abrogated the downregulatory effect of ANXA1 on IL-1*β* production. These results indicate that in human CL due to *L. braziliensis* the main receptor for ANXA1 is FPR2.

Since it has already been documented that human monocytes-derived macrophages have an innate capacity to kill *L. braziliensis* through increased production of reactive oxygen species, we assessed the infection rate and the average number of amastigotes per cell in these cultures and demonstrated that, regardless of the inflammatory modulation conditions, macrophages showed a reduction in these metrics from 4 to 48 hours, confirming their innate microbicidal capacity, independent of low levels of IL-1*β* (46, 47). Although in mice model it has been documented that IL-1*β* production is necessary for *Leishmania* parasite killing, studies using human monocytes/macrophages did not find an effect of IL-1*β* in *Leishmania* killing (23, 24). These results suggest the decrease of IL-1*β* production induced by ANXA1 would benefit the patients without affecting the ability macrophages to kill parasites. The data from this study strengthens the role of ANXA1 as a modulator of the inflammatory response in CL, highlighting its ability to regulate IL-1*β* production in both systemic and lesion sites without interfering with IL-10 levels or parasite killing. This selective action suggests that ANXA1 contributes to controlling excessive inflammation, characteristic of the disease, without compromising essential regulatory mechanisms.

From a clinical perspective, these findings position ANXA1 as a potential adjuvant in treating CL lesions, able of modulating the inflammatory response in a controlled manner, favoring tissue resolution without impairing parasite killing. Future clinical studies are needed to validate the ANXA1 role as a biomarker or therapeutic agent in immunomodulatory management strategies for CL.

## Data Availability

The original contributions presented in the study are included in the article/supplementary material. Further inquiries can be directed to the corresponding author.

## References

[1] Jirmanus L Glesby MJ Guimaraes LH Lago E Rosa ME MaChado PR. Epidemiological and clinical changes in american tegumentary leishmaniasis in an area of leishmania (viannia) Braziliensis transmission over a 20-year period. Am J Trop Med hygiene. (2012) 86:426. doi: 10.4269/ajtmh.2012.11-0378, PMID: 22403312 PMC3284357

[2] Saldanha MG Queiroz A MaChado PRL De Carvalho LP Scott P de Carvalho Filho EM. Characterization of the histopathologic features in patients in the early and late phases of cutaneous leishmaniasis. Am J Trop Med Hygiene. (2017) 96:645. doi: 10.4269/ajtmh.16-0539, PMID: 28115669 PMC5361539

[3] Bittencourt A Barral A. Evaluation of the histopathological classifications of american cutaneous and mucocutaneous leishmaniasis. Memo´rias do Instituto Oswaldo Cruz. (1991) 86:51–6. doi: 10.1590/S0074-02761991000100009, PMID: 1842401

[4] Faria DR Gollob KJ Barbosa J Jr. Schriefer A MaChado PR Lessa H. Decreased in *situ* expression of interleukin-10 receptor is correlated with the exacerbated inflammatory and cytotoxic responses observed in mucosal leishmaniasis. Infection Immun. (2005) 73:7853–9. doi: 10.1128/IAI.73.12.7853-7859.2005, PMID: 16299275 PMC1307048

[5] Saldanha MG Pagliari C Queiroz A MaChado PRL Carvalho L Scott P. Tissue damage in human cutaneous leishmaniasis: correlations between inflammatory cells and molecule expression. Front Cell Infection Microbiol. (2020) 10:355. doi: 10.3389/fcimb.2020.00355, PMID: 32766167 PMC7381142

[6] Unger A O’Neal S MaChado PR Guimarães LH Morgan DJ Schriefer A. Association of treatment of american cutaneous leishmaniasis prior to ulcer development with high rate of failure in northeastern Brazil. Am J Trop Med hygiene. (2009) 80:574. doi: 10.4269/ajtmh.2009.80.574, PMID: 19346378 PMC3557504

[7] Costa RS Carvalho LP Campos TM Magalhães AS Passos ST Schriefer A. Early cutaneous leishmaniasis patients infected with leishmania Braziliensis express increased inflammatory responses after antimony therapy. J Infect Dis. (2018) 217:840–50. doi: 10.1093/infdis/jix627, PMID: 29216363 PMC5853895

[8] Flower RJ Rothwell NJ. Lipocortin-1: cellular mechanisms and clinical relevance. Trends Pharmacol Sci. (1994) 15:71–6. doi: 10.1016/0165-6147(94)90281-X, PMID: 8184489

[9] Perretti M D’acquisto F. Annexin a1 and glucocorticoids as effectors of the resolution of inflammation. Nat Rev Immunol. (2009) 9:62–70. doi: 10.1038/nri2470, PMID: 19104500

[10] Perretti M Leroy X Bland EJ Montero-Melendez T. Resolution pharmacology: opportunities for therapeutic innovation in inflammation. Trends Pharmacol Sci. (2015) 36:737–55. doi: 10.1016/j.tips.2015.07.007, PMID: 26478210

[11] Flower RJ Blackwell G. Anti-inflammatory steroids induce biosynthesis of a phospholipase a2 inhibitor which prevents prostaglandin generation. Nature. (1979) 278:456–9. doi: 10.1038/278456a0, PMID: 450050

[12] Gavins FN Yona S Kamal AM Flower RJ Perretti M. Leukocyte antiadhesive actions of annexin 1: Alxr-and fpr-related anti-inflammatory mechanisms. Blood J Am Soc Hematol. (2003) 101:4140–7. doi: 10.1182/blood-2002-11-3411, PMID: 12560218

[13] Hannon R Croxtall JD Getting SJ Roviezzo F Yona S Paul-Clark MJ. Aberrant inflammation and resistance to glucocorticoids in annexin 1-/- mouse. FASEB J. (2003) 17:253–5., PMID: 12475898 10.1096/fj.02-0239fje

[14] La M D’Amico M Bandiera S Filippo CD Oliani SM Gavins FN. Annexin 1 peptides protect against experimental myocardial ischemia-reperfusion: analysis of their mechanism of action. FASEB J. (2001) 15:2247–56. doi: 10.1096/fj.01-0196com, PMID: 11641252

[15] Girol AP Mimura KK Drewes CC Bolonheis SM Solito E Farsky SH. Anti-inflammatory mechanisms of the annexin a1 protein and its mimetic peptide ac2–26 in models of ocular inflammation *in vivo* and in *vitro* . J Immunol. (2013) 190:5689–701. doi: 10.4049/jimmunol.1202030, PMID: 23645879

[16] Guido BC Zanatelli M Tavares-de Lima W Oliani SM Damazo AS. Annexin-a1 peptide down-regulates the leukocyte recruitment and up-regulates interleukin-10 release into lung after intestinal ischemia-reperfusion in mice. J Inflammation. (2013) 10:1–10. doi: 10.1186/1476-9255-10-10, PMID: 23497133 PMC3608250

[17] Amorim CF Novais FO Nguyen BT Misic AM Carvalho LP Carvalho EM. Variable gene expression and parasite load predict treatment outcome in cutaneous leishmaniasis. Sci Trans Med. (2019) 11:eaax4204. doi: 10.1126/scitranslmed.aax4204, PMID: 31748229 PMC7068779

[18] Farias Amorim C O. Novais F Nguyen BT Nascimento MT Lago J Lago AS. Localized skin inflammation during cutaneous leishmaniasis drives a chronic, systemic ifn-*γ* signature. PloS Negl Trop Dis. (2021) 15:e0009321., PMID: 33793565 10.1371/journal.pntd.0009321 PMC8043375

[19] Cupolillo E Grimaldi G Jr. Momen H. A general classification of new world leishmania using numerical zymotaxonomy. Am J Trop Med Hygiene. (1994) 50:296–311. doi: 10.4269/ajtmh.1994.50.296, PMID: 8147488

[20] Medzhitov R Janeway JC. The toll receptor family and microbial recognition. Trends Microbiol. (2000) 8:452–6. doi: 10.1016/S0966-842X(00)01845-X, PMID: 11044679

[21] Polari LP Carneiro PP Macedo M MaChado PR Scott P Carvalho EM. Leishmania Braziliensis infection enhances toll-like receptors 2 and 4 expression and triggers tnf-*α* and il-10 production in human cutaneous leishmaniasis. Front Cell infection Microbiol. (2019) 9:120. doi: 10.3389/fcimb.2019.00120, PMID: 31119102 PMC6507514

[22] Nascimento MT Franca M Carvalho AM Amorim CF Peixoto F Beiting D. Inhibition of gamma-secretase activity without interfering in notch signalling decreases inflammatory response in patients with cutaneous leishmaniasis. Emerging Microbes Infections. (2021) 10:1219–26. doi: 10.1080/22221751.2021.1932608, PMID: 34009107 PMC8676695

[23] Lima-Junior DS Costa DL Carregaro V Cunha LD Silva AL Mineo TW. Inflammasome-derived il-1*β* production induces nitric oxide–mediated resistance to leishmania. Nat Med. (2013) 19:909–15. doi: 10.1038/nm.3221, PMID: 23749230

[24] Santos D Campos TM Saldanha M Oliveira SC Nascimento M Zamboni DS. Il-1*β* production by intermediate monocytes is associated with immunopathology in cutaneous leishmaniasis. J Invest Dermatol. (2018) 138:1107–15. doi: 10.1016/j.jid.2017.11.029, PMID: 29246797 PMC5912958

[25] Ferrari CC Godoy MCP Tarelli R Chertoff M Depino AM Pitossi FJ. Progressive neurodegeneration and motor disabilities induced by chronic expression of il-1*β* in the substantia nigra. Neurobiol Dis. (2006) 24:183–93. doi: 10.1016/j.nbd.2006.06.013, PMID: 16901708

[26] Dinarello CA Simon A van der Meer JW. Treating inflammation by blocking interleukin-1 in a broad spectrum of diseases. Nat Rev Drug Discov. (2012) 11:633–52. doi: 10.1038/nrd3800, PMID: 22850787 PMC3644509

[27] Charmoy M Hurrell BP Romano A Lee SH Ribeiro-Gomes F Riteau N. The nlrp3 inflammasome, il-1*β*, and neutrophil recruitment are required for susceptibility to a nonhealing strain of leishmania major in c57bl/6 mice. Eur J Immunol. (2016) 46:897–911. doi: 10.1002/eji.201546015, PMID: 26689285 PMC4828310

[28] Ji J Sun J Soong L. Impaired expression of inflammatory cytokines and chemokines at early stages of infection with leishmania amazonensis. Infection Immun. (2003) 71:4278–88. doi: 10.1128/IAI.71.8.4278-4288.2003, PMID: 12874303 PMC166010

[29] Koutsoni OS Barhoumi M Guizani I Dotsika E. New insights on the adjuvant properties of the leishmania infantum eukaryotic initiation factor. J Immunol Res. (2019) 2019:9124326. doi: 10.1155/2019/9124326, PMID: 31183394 PMC6515109

[30] Darzi F Davoudian R Nateghi Rostami M. Differential inflammatory responses associated with leishmania major and l tropica in culture. Parasite Immunol. (2021) 43:e12841. doi: 10.1111/pim.12841, PMID: 33914948

[31] Carvalho AM Costa RS Lago A Bacellar O Beiting DP Scott P. *In situ* versus systemic immune response in the pathogenesis of cutaneous leishmaniasis. Pathogens. (2024) 13:199. doi: 10.3390/pathogens13030199, PMID: 38535542 PMC10975199

[32] Carvalho AM Guimarães LH Costa R Saldanha MG Prates I Carvalho LP. Impaired th1 response is associated with therapeutic failure in patients with cutaneous leishmaniasis caused by leishmania Braziliensis. J Infect Dis. (2021) 223:527–35. doi: 10.1093/infdis/jiaa374, PMID: 32620011 PMC7881333

[33] Oliveira WN Ribeiro LE Schrieffer A MaChado P Carvalho EM Bacellar O. The role of inflammatory and anti-inflammatory cytokines in the pathogenesis of human tegumentary leishmaniasis. Cytokine. (2014) 66:127–32. doi: 10.1016/j.cyto.2013.12.016, PMID: 24485388 PMC4047562

[34] Quirino GFdS. A participação do inflamassoma de NLRP3 no desenvolvimento de Leishmaniose cutaˆnea localizada e difusa (2019). Universidade de Sa˜o Paulo, Ribeirão Preto.

[35] Zamboni DS Sacks DL. Inflammasomes and leishmania: in good times or bad, in sickness or in health. Curr Opin Microbiol. (2019) 52:70–6. doi: 10.1016/j.mib.2019.05.005, PMID: 31229882 PMC6910972

[36] Tylek K Trojan E Regulska M Lacivita E Leopoldo M Basta-Kaim A. Formyl peptide receptor 2, as an important target for ligands triggering the inflammatory response regulation: a link to brain pathology. Pharmacol Rep. (2021) 73:1004–19. doi: 10.1007/s43440-021-00271-x, PMID: 34105114 PMC8413167

[37] Galva˜o I de Carvalho RV Vago JP Silva AL Carvalho TG Antunes MM. The role of annexin a1 in the modulation of the nlrp3 inflammasome. Immunology. (2020) 160:78–89. doi: 10.1111/imm.13184, PMID: 32107769 PMC7160667

[38] Sanches JM Branco LM Duarte GHB Oliani SM Bortoluci KR Moreira V. Annexin a1 regulates nlrp3 inflammasome activation and modifies lipid release profile in isolated peritoneal macrophages. Cells. (2020) 9:926. doi: 10.3390/cells9040926, PMID: 32283822 PMC7226734

[39] Sasso GRDS Cerri PS Sasso-Cerri E Simões MJ Gil CD Florencio-Silva R. Possible role of annexin a1/fpr2 pathway in cox2/nlrp3 inflammasome regulation in alveolar bone cells of estrogen-deficient female rats with diabetes mellitus. J Periodontology. (2024) 95:749–63. doi: 10.1002/JPER.23-0530, PMID: 37987258

[40] Correia-Silva RD Corrêa MP de Castro ME Almeida JS D'Ávila SC Oliani SM. Regulatory role of annexin a1 in nlrp3 inflammasome activation in atopic dermatitis: insights from keratinocytes in human and murine studies. J Mol Med. (2025), 1–17. doi: 10.1007/s00109-025-02529-w, PMID: 40100418

[41] Galva˜o I Vago JP Barroso LC Tavares LP Queiroz-Junior CM Costa VV. Annexin a1 promotes timely resolution of inflammation in murine gout. Eur J Immunol. (2017) 47:585–96. doi: 10.1002/eji.201646551, PMID: 27995621

[42] Perretti M Ingegnoli F Wheller SK Blades MC Solito E Pitzalis C. Annexin 1 modulates monocyte-endothelial cell interaction *in vitro* and cell migration *in vivo* in the human scid mouse transplantation model. J Immunol. (2002) 169:2085–92. doi: 10.4049/jimmunol.169.4.2085, PMID: 12165536 PMC4340507

[43] Ricotta TQN Dos Santos LM Oliveira LG Souza-Testasicca MC Nascimento FC Vago JP. Annexin a1 improves immune responses and control of tissue parasitism during leishmania amazonensis infection in balb/c mice. Biomedicine Pharmacotherapy. (2024) 172:116254. doi: 10.1016/j.biopha.2024.116254, PMID: 38340398

[44] Han P-F Che X-D Li H-Z Gao Y-Y Wei X-C Li P-C. Annexin a1 involved in the regulation of inflammation and cell signaling pathways. Chin J Traumatology. (2020) 23:96–101. doi: 10.1016/j.cjtee.2020.02.002, PMID: 32201231 PMC7156956

[45] Oliveira LG Souza-Testasicca MC Vago JP Figueiredo AB Canavaci A Perucci LO. Annexin a1 is involved in the resolution of inflammatory responses during leishmania Braziliensis infection. J Immunol. (2017) 198:3227–36. doi: 10.4049/jimmunol.1602028, PMID: 28289158

[46] Peixoto F Nascimento MT Costa R Silva J Renard G Guimarães LH. Evaluation of the ability of miltefosine associated with topical gm-csf in modulating the immune response of patients with cutaneous leishmaniasis. J Immunol Res. (2020) 2020:2789859. doi: 10.1155/2020/2789859, PMID: 32851099 PMC7439779

[47] Novais FO Carvalho AM Clark ML Carvalho LP Beiting DP Brodsky IE. Cd8+ t cell cytotoxicity mediates pathology in the skin by inflammasome activation and il-1*β* production. PloS Pathog. (2017) 13:e1006196. doi: 10.1371/journal.ppat.1006196, PMID: 28192528 PMC5325592

